# Solid part size is an important predictor of nodal metastasis in lung cancer with a subsolid tumor

**DOI:** 10.1186/s12890-018-0709-2

**Published:** 2018-09-10

**Authors:** Jun Yeun Cho, Cho Sun Leem, Youlim Kim, Eun Sun Kim, Sang Hoon Lee, Yeon Joo Lee, Jong Sun Park, Young-Jae Cho, Jae Ho Lee, Choon-Taek Lee, Ho Il Yoon

**Affiliations:** 1 Division of Pulmonary and Critical Care Medicine, Department of Internal Medicine, Seoul National University Bundang Hospital, 82, Gumi-ro 173 Beon-gil, Bundang-gu, Seongnam-si, Gyeonggi-do 13620 Republic of Korea; 2 Department of Internal Medicine, Seoul National University College of Medicine, Seoul, Republic of Korea

**Keywords:** Risk factor, Non-small cell lung Cancer, Lymphatic metastasis

## Abstract

**Background:**

Candidates for preoperative or intraoperative nodal assessment among patients with non-small cell lung cancer (NSCLC) manifesting as a subsolid tumor are not established. The present study was conducted to demonstrate the distribution of nodal metastasis rate according to newly proposed T categories for subsolid tumors, and we further aimed to identify radiologic parameters that can be predictive of nodal metastasis.

**Methods:**

We retrospectively reviewed cases of NSCLC manifesting as a subsolid tumor in computed tomography scans in a university-affiliated tertiary hospital between April 2013 and August 2016. All patients underwent mediastinal lymph node dissection during resection surgery. Multivariate analysis was performed among clinical and radiologic parameters.

**Results:**

Of the 269 eligible patients, T-categories were classified as cTis (*n* = 23, 8.6%), cT1 (*n* = 203, 75.5%), and cT2 (*n* = 43, 16.0%). Ten patients (3.7%) had nodal metastasis: pN1 (*n* = 5, 1.9%), pN2 (*n* = 5, 1.9%). Nodal metastasis was not observed in tumors with a solid part ≤1.0 cm (cT1mi and cT1a) or in nonsolid tumors ≤3.0 cm (cTis). The nodal metastasis rate in cT1b, cT1c, and cT2 tumors was 6.1% (4/65), 8.3% (1/12), and 11.7% (5/43), respectively. Multivariate analysis showed that a solid part size > 1.5 cm [odds ratio, 5.89; 95% confidence interval, 1.25–27.68, *p* = 0.025] was significantly associated with nodal metastasis.

**Conclusions:**

We observed nodal metastasis from cT1b tumors (solid part size > 1 cm) among proposed T categories for subsolid tumors and a solid part size is an important radiologic parameter predictive of nodal metastasis in NSCLC manifesting as a subsolid tumor. Considering the low rate of nodal metastasis, pathologic nodal assessment may be unnecessary in early T category tumors with a small solid part size.

## Background

The considerable increase in lung cancer screening has recently led to issues such as an increased number of lung nodules discovered via computed tomography (CT) and the management of these lesions. The subsolid nodule, defined as a well-demarcated lung lesion containing a ground-glass opacity, exhibits different behaviors from a solid nodule and accordingly, has garnered much attention. In 2013, the Fleischner Society recommendations emphasized that from a management perspective, both pure and part-solid ground-glass nodules are best considered as a category separate from purely solid lesions [1]. Subsequently, many studies have investigated subsolid nodules, and consequently, the recently proposed eighth tumor-node-metastasis staging system includes more detailed T1 categories of subsolid nodules, compared to previous versions [2].

Accurate nodal staging is fundamental in diagnosing and treating non-small cell lung cancer (NSCLC). Generally, the nodal metastasis rate in patients with NSCLC manifesting as a subsolid nodule has been known to be low [3–5]. Therefore, it is questionable whether preoperative or intraoperative nodal assessment is needed. Recently updated National Comprehensive Cancer Network guidelines state that preoperative pathologic mediastinal evaluation is optional for solid tumors < 1.0 cm and purely non-solid tumors < 3.0 cm with radiologic negative mediastinum [6] because of the low rate of mediastinal metastasis. Intraoperative nodal assessment in this situation is also controversial, and there is no consensus. Currently, many clinicians conduct intraoperative nodal assessment based on their experiences.

Given this background, the present study was conducted to demonstrate the distribution of nodal metastasis rate according to newly proposed T categories for subsolid tumors. For selecting candidates for preoperative or intraoperative nodal assessment, we further aimed to identify radiologic parameters that can be predictive of nodal metastasis.

## Methods

We selected patients with surgically resected NSCLC manifesting as a subsolid tumor on CT scans in a university affiliated-tertiary hospital from April 2013 to August 2016. All patients underwent mediastinal lymph node dissection (MLND) during resection surgery. The patient’s clinical data, radiologic features, and pathologic results were retrospectively reviewed.

The definitions of nodal zone and nodal station were based on the International Association of the Study of Lung Cancer (IASLC) lymph node map [7]. The pathologic diagnoses were based on the 2011 IASLC classification [8]. Real-time endobronchial ultrasound (EBUS) was performed in selected patients. Rapid on-site evaluation was not conducted during EBUS procedure.

We described radiologic features of a subsolid tumor based on expert thoracic radiologists’ reports. In the present study, we defined a subsolid tumor as a mass (> 3 cm) or nodule (≤3 cm) that contained ground-glass lesions on CT images. The total tumor (including ground glass portion around solid part) and solid part size were measured as the maximum diameters on the lung window setting. Subsolid tumors less than or equal to 3 cm were classified according to recently proposed T categories [2].T categories were classified by the total tumor and solid part size; cTis (total tumor size of 0.6–3.0 cm with no solid part), cT1 (total tumor size of 0.6–3.0 cm with solid part size 0.6–3.0 cm) and cT2 (total tumor size of 3.0–7.0 cm with any solid part size).

If a total tumor measured 3 cm to 7 cm, it was categorized as cT2 regardless of the solid part size. Cases with multiple tumors were excluded. A tumor was considered central if it was visualized within the inner third of the lung field or abutted mediastinal structures on CT or positron emission tomography (PET) images. Radiologic N staging was determined from CT scans with or without PET findings. Lymph nodes with shortest diameters of > 1 cm on CT and/or a maximum standardized uptake value > 2.5 on PET were considered metastatic. Pathologic N stage was determined by the final pathologic report after surgery.

All data are presented as mean values (± standard deviations) for continuous variables and numbers (percentages) for categorical variables. Data were compared between defined groups using the Mann–Whitney test for continuous variables and Fisher’s exact test for categorical variables. A linear-by-linear association was defined using Pearson’s coefficient and performed to test the trends of ordinal scales in categorical variables.

Significant variables from clinical and radiologic data identified in the univariate analysis were used for multivariate analysis to elucidate predictive factors of nodal metastasis. SPSS 22.0 (IBM, Armonk, NY, USA) was used for the statistical analyses. A *p* value < 0.05 was considered statistically significant.

## Results

A total of 269 eligible patients were identified, and their baseline characteristics are summarized in Table 1. The patients had a mean age of 62.4 ± 10.4 years. One hundred fifteen patients (42.8%) were male, and 90 (33.5%) were former or current smokers. Fourteen (5.2%) patients had a centrally located tumor. The tumors were categorized into three groups: cTis (*n* = 25, 8.9%), cT1 (*n* = 212, 75.7%), and cT2 (*n* = 43, 15.4%). There were no cases presenting as a nonsolid tumor less than 0.5 cm. Ninety-nine (36.8%) patients underwent EBUS prior to resection surgery, and 228 patients (84.8%) underwent lobectomy. The most frequent tissue pathology was invasive adenocarcinoma (*n* = 235, 84.8%). We identified 10 patients with nodal metastasis: pN1 (*n* = 5, 1.9%) and pN2 (*n* = 5, 1.9%).

**Table 1:** Clinical, radiologic and pathologic characteristics of patients

Characteristic	Total (*n* = 269)
Age, years	62.4 ± 10.4
Male sex	115 (42.8)
Former/Current smoker	90 (33.5)
Previous extra-thoracic malignancy	34 (12.6)
Previous lung cancer	6 (2.2)
Tumor centrality	14 (5.2)
Radiologic N stage	
N0	227 (84.4)
N1–2	42 (15.6)
T categories for subsolid tumor^a^	
cTis	23 (8.6)
cT1	203 (75.5)
cT2	43 (16.0)
EBUS	99 (36.8)
Operation extent	
Segmentectomy	41 (15.2)
Lobectomy	228 (84.8)
Pathology	
AAH/AIS	2 (0.7)
MIA	29 (10.8)
Invasive ADC	235 (87.4)
Invasive mucinous ADC	3 (1.1)
Pathologic N stage^b^	
N0	259 (96.3)
N1	5 (1.9)
N2	5 (1.9)

Data are presented as n (%) or mean ± standard deviation

*AAH* atypical adenomatous hyperplasia, *ADC* adenocarcinoma, *AIS* adenocarcinoma in situ, *EBUS* endobronchial ultrasound, *MIA* minimally invasive adenocarcinoma

^a^T categories were classified by the total tumor size (cm) and solid part size (cm). Total tumor size was defined as a size including ground glass around the solid part; cTis (total tumor size of 0.6–3.0 cm with no solid part), cT1 (total tumor size of 0.6–3.0 cm with solid part size 0.6–3.0 cm) and cT2 (total tumor size of 3.0–7.0 cm with any solid part size)

^b^Pathologic N stage was determined by the final pathologic report after surgery

Patients with more advanced clinical T category frequently underwent EBUS and lobectomy (Table 2). In cT1 tumors, the pN1 and pN2 rates were 1.5 and 1.0%, respectively, whereas there was no nodal metastasis in cTis tumors. In cT2 tumors, the pN1 and N2 rates were 4.7 and 7.0%, respectively. More advanced T tumors had significantly more advanced nodal metastasis. In addition, invasive adenocarcinomas were frequently diagnosed in advanced T tumors.

**Table 2:** Differences between cTis, cT1 and cT2 tumors

Variables	cTis (*n* = 23)	cT1 (*n* = 203)	cT2 (*n* = 43)	*p* Value
EBUS				0.000
Not performed	21 (91.3)	134 (66.0)	15 (34.9)	
Performed	2 (8.7)	69 (34.0)	28 (65.1)	
Operation extent				0.009
Segmentectomy	5 (21.7)	36 (17.7)	0 (0.0)	
Lobectomy	18 (78.3)	167 (82.3)	43 (100.0)	
Pathologic N stage				0.039
N0	23 (100.0)	198 (97.5)	38 (88.4)	
N1	0 (0.0)	3 (1.5)	2 (4.7)	
N2	0 (0.0)	2 (1.0)	3 (7.0)	
Pathology				0.002
AAH/AIS	0 (0.0)	2 (1.0)	0 (0.0)	
MIA	7 (30.4)	22 (10.8)	0 (0.0)	
Invasive ADC	16 (69.6)	176 (86.7)	43 (100.0)	
Invasive mucinous ADC	0 (0.0)	3 (1.5)	0 (0.0)	

Data are presented as n (%)

*AAH* atypical adenomatous hyperplasia, *ADC* adenocarcinoma, *AIS* adenocarcinoma in situ, *EBUS* endobronchial ultrasound, *MIA* minimally invasive adenocarcinoma

The distribution of nodal metastasis in tumors classified by the proposed clinical T categories is detailed in Table 3. Nodal metastasis was observed in tumors in which the solid part was > 1.0 cm or total tumor size > 3.0 cm. There was no nodal metastasis in cTis, cT1mi, and cT1a tumors. The nodal metastasis rates in cT1b, cT1c, and cT2 tumors were 6.1% (4/65), 8.3% (1/12), and 11.7% (5/43), respectively. The mean solid part size in cT2 tumors (2 cases were nonsolid tumors) was 2.0 ± 1.0 cm.

**Table 3:** Distribution of nodal metastasis in tumors classified by proposed clinical T categories^a^

Categories	Total tumor Size^b^ (cm)	Solid part Size (cm)	pN0	pN1	pN2
cTis	0.6–3.0	0	23 (100.0)	0 (0.0)	0 (0.0)
cT1mi	≤3.0	≤0.5	46 (100.0)	0 (0.0)	0 (0.0)
cT1a	0.6–3.0	0.6–1.0	80 (100.0)	0 (0.0)	0 (0.0)
cT1b	1.1–3.0	1.1–2.0	61 (93.8)	3 (4.6)	1 (1.5)
cT1c	2.1–3.0	2.1–3.0	11 (91.7)	0 (0.0)	1 (8.3)
cT2^c^	3.0–7.0	Any	38 (88.4)	2 (4.7)	3 (7.0)

^a^The nodal metastasis rate was significantly different among classified T categories (*P* = 0.033)

^b^Defined as a size including ground glass portion around the solid part

^c^Two cases were nonsolid tumors, and the mean solid part size was 2.0 ± 1.0 cm

The detailed information of patients with nodal metastasis is presented in Table 4. All patients had a solid part exceeding 1 cm. Only one patient had a centrally located tumor. Most cases of metastatic N1 involved a peribronchial node, and 3 of 5 cases of metastatic N2 involved a subcarinal node. Tumors with nodal metastasis were invasive adenocarcinoma with acinar, papillary, or solid subtype. Four patients with pN2 underwent EBUS prior to surgery. One patient (patient number 9) was diagnosed with pN2 disease by EBUS, and subsequently underwent surgery due to single N2. Other cases (patient number 7, 8, 10) were diagnosed with pN2 disease by surgery.

**Table 4:** Detailed characteristics of patients with nodal involvement^a^

Patient number	Total tumor size	Solid part size	T stage	Central Tumor	N Stage (CT)	N Stage (PET)	EBUS	pN stage	Involved N1	Involved N2	Pathology/Subtype
1	3.0	1.7	cT1b	No	0	0	Yes	pN1	Interlobar	–	ADC/papillary
2	1.8	1.4	cT1b	No	0	0	Yes	pN1	Peribronchial	–	ADC/solid
3	4.1	2.3	cT2	No	0	2	Yes	pN1	Peribronchial	–	ADC/acinar
4	3.2	3.1	cT2	No	0	0	Yes	pN1	Peribronchial	–	ADC/papillary
5	2.7	1.2	cT1b	No	0	0	Yes	pN1	Peribronchial	–	ADC/acinar
6	3.7	2.8	cT2	No	0	0	No	pN2	Peribronchial	2,3,4	ADC/papillary
7	3.2	1.3	cT2	Yes	0	0	Yes	pN2	–	6	ADC/acinar
8	5.4	2.0	cT2	No	0	2	Yes	pN2	Peribronchial	7	ADC/acinar
9	2.7	2.6	cT1c	No	1	0	Yes	pN2	Peribronchial	7	ADC/acinar
10	2.4	1.8	cT1b	No	0	0	Yes	pN2	–	7	ADC/papillary

*ADC* adenocarcinoma, *CT* computed tomography, *EBUS* endobronchial ultrasound, *PET* positron emission tomography

^a^Four patients with pN2 underwent EBUS prior to surgery. One patient (patient number 9) was diagnosed with pN2 disease by EBUS, and subsequently underwent surgery due to single N2. Other cases (patient number 7, 8, 10) were diagnosed with pN2 disease by surgery

We performed a multivariate analysis to identify the risk factors predictive of nodal metastasis (Table 5). The multivariate analysis included variables that were identified as significant in the univariate analysis. A solid part size > 1.5 cm [odds ratio (OR), 5.89; 95% confidence interval (CI), 1.25–27.68, *p* = 0.025] was found to associate significantly with nodal metastasis.

**Table 5:** Risk factors predictive of nodal metastasis

	Univariate		Multivariate		
Variables	OR	95% CI	OR	95% CI	*p* Value
Total tumor size > 3 cm	5.81	1.61–21.05	2.46	0.57–10.50	0.226
Solid part size > 1.5 cm	8.66	2.17–34.57	5.89	1.25–27.68	0.025
Tumor centrality	2.10	0.25–17.87			
Radiologic nodal metastasis	2.42	0.60–9.75			

*CI* confidence interval, *OR* odds ratio

## Discussion

In our cohort, the nodal metastasis rate was 3.7% (1.9% with both pN1 and pN2). Among part-solid nodules (cT1a-cT1c), the pN1 and pN2 rates were 1.5 and 1.0%, respectively, whereas there was no nodal metastasis in cTis (nonsolid nodule ≤3 cm). These findings of low incidence of nodal metastasis are consistent with previous studies [4, 5, 9].

When tumors were classified by the proposed new T categories, any nodal metastasis was observed from cT1b to cT2 tumors, in which the solid part size was > 1.0 cm or total tumor size > 3.0 cm. In the final pathologic reports, patients with nodal metastasis had adenocarcinoma with invasive subtypes. There was no early stage adenocarcinoma (e.g., adenocarcinoma in situ (AIS), minimally invasive adenocarcinoma (MIA), lepidic predominant adenocarcinoma (LPA)). These findings suggest that preoperative pathologic nodal evaluation or extended lymph node assessment might be unnecessary when early stage adenocarcinomas are suspected by radiologic features.

It is debatable whether extended lymph node assessment should be performed in patients with early stage NSCLC. As noted earlier, the incidence of nodal metastasis in NSCLCs manifesting as a subsolid tumor is lower than in NSCLCs with pure solid tumor. Furthermore, the extent of intraoperative node assessment (e.g., selective sampling vs. systematic dissection) in NSCLC patients remains controversial [10]. Although more extended node dissection has positive effects on clinical outcomes [11, 12], it is important to consider various procedure-related morbidities. Flores et al. compared 151 cases of NSCLC (subsolid nodules) with MLND and 52 cases of NSCLC without MLND [13]. They observed no differences in survival and asserted that performing MLND is not mandatory when screen-diagnosed NSCLC manifests as a subsolid nodule.

Currently available guidelines recommend that preoperative pathologic mediastinal evaluation (e.g., EBUS) should be considered regarding tumor size, radiologic N status, and tumor centrality [14, 15]. However, they do not suggest recommendations exclusively for subsolid tumors. In the present study, multivariate regression analysis showed that solid part size (> 1.5 cm) is predictive of nodal metastasis. Previous studies focused on importance of solid consistency for predicting nodal metastasis in NSCLCs. Koike et al. suggested 89% solid consistency (proportion of solid part size in total tumor size including ground glass) as a cutoff value to predict mediastinal metastasis in clinical IA NSCLCs [16]. Gao et al. reported that an occult N2 risk was lower in tumors with a ground glass component than in tumors without, among T1–2 N0 NSCLCs determined by PET CT [17]. Ye et al. asserted that ground glass status (part solid or pure solid vs nonsolid) is more accurate predictor than tumor diameter in clinical IA adenocarcinoma [4]. However, these studies included numbers of pure solid tumors, not only subsolid tumors. Our study’s strength is that only cases of NSCLC manifesting as subsolid tumors were included. This is an important finding because solid part size can be a determinant radiologic criterion when preoperative pathologic nodal assessments are performed.

Tumor size has been considered an important risk factor for predicting nodal metastasis, and preoperative pathologic nodal assessment was considered in cases of tumor exceeding 3 because of low negative predictive value of PET-CT for detecting mediastinal nodal metastasis [18, 19]. However, total tumor size including ground glass was not a significant predictive factor (Table 5). The relatively small number of cT2 cases might have contributed to this result. Moreover, data from Table 2 shows that the nodal metastasis rate was significantly higher in cT2 tumors than in cTis or cT1 tumors. We believe that total tumor size is still worthy as an important predictive radiologic factor, as well as solid part size.

In our study, we did not find an association between tumor centrality and nodal metastasis, although tumor location has been considered an important factor related to NSCLC. In a study by Lee et al. [20] of 221 patients with clinical IA NSCLC with a radiologically negative mediastinum, the frequency of central tumor location was 23%, with a higher incidence of pN2 disease relative to peripherally located tumors. However, only 5.2% of tumors in the present study were centrally located, and primary lung adenocarcinomas were generally located peripherally. Moreover, the term “tumor centrality” is vague even among radiologists. Hence, the ability of tumor centrality to predict nodal metastasis should be evaluated in future studies.

There are some limitations in our study. First, because of the retrospective design nature, selection bias may have affected the results. EBUS and lobectomy were performed more frequently in more advanced T categories (Table 2). Therefore, it is assumed that clinicians did not actively conduct preoperative or intraoperative nodal assessment in patients with early stage NSCLCs (AIS, MIA, and LPA). Indeed, we identified 376 surgically resected NSCLCs manifesting as a subsolid tumor during the study period and excluded 107 patients who did not undergo MLND. Second, the actual nodal metastasis rate was low because we included only subsolid tumors. Hence, logistic regression analysis showed widened confidence intervals for the odds ratios, subsequently indicating statistically weak data. Future studies correcting these limitations are needed.

## Conclusions

Among the proposed T categories for subsolid nodules, we observed nodal metastasis from cT1b, in which the solid part size exceeded 1 cm. The nodal metastasis rate was 3.7%, and solid part size is an important radiologic parameter predictive of nodal metastasis in NSCLC manifesting as a subsolid tumor. Considering the low rate of nodal metastasis, pathologic nodal assessment may be unnecessary in early T category tumors with a small solid part size.

## Abbreviations

AIS: Adenocarcinoma in situ
CI: Confidence interval
CT: Computed tomography
EBUS: Endobronchial ultrasound
IASLC: International Association of the Study of Lung Cancer
LPA: Lepidic predominant adenocarcinoma
MIA: Minimally invasive adenocarcinoma
MLND: Mediastinal lymph node dissection
NSCLC: Non-small cell lung cancer
OR: Odds ratio
PET: Positron emission tomography
